# Supplementary material for: Immunoinformatics mapping of potential epitopes in SARS-CoV-2 structural proteins
Source: PLoS One. 2021 Nov 15;16(11):e0258645. doi: 10.1371/journal.pone.0258645 (PMC8592446; doi:10.1371/journal.pone.0258645)
Supplement: S1 File — (PPTX) [file pone.0258645.s001.pptx]

## Slide 1
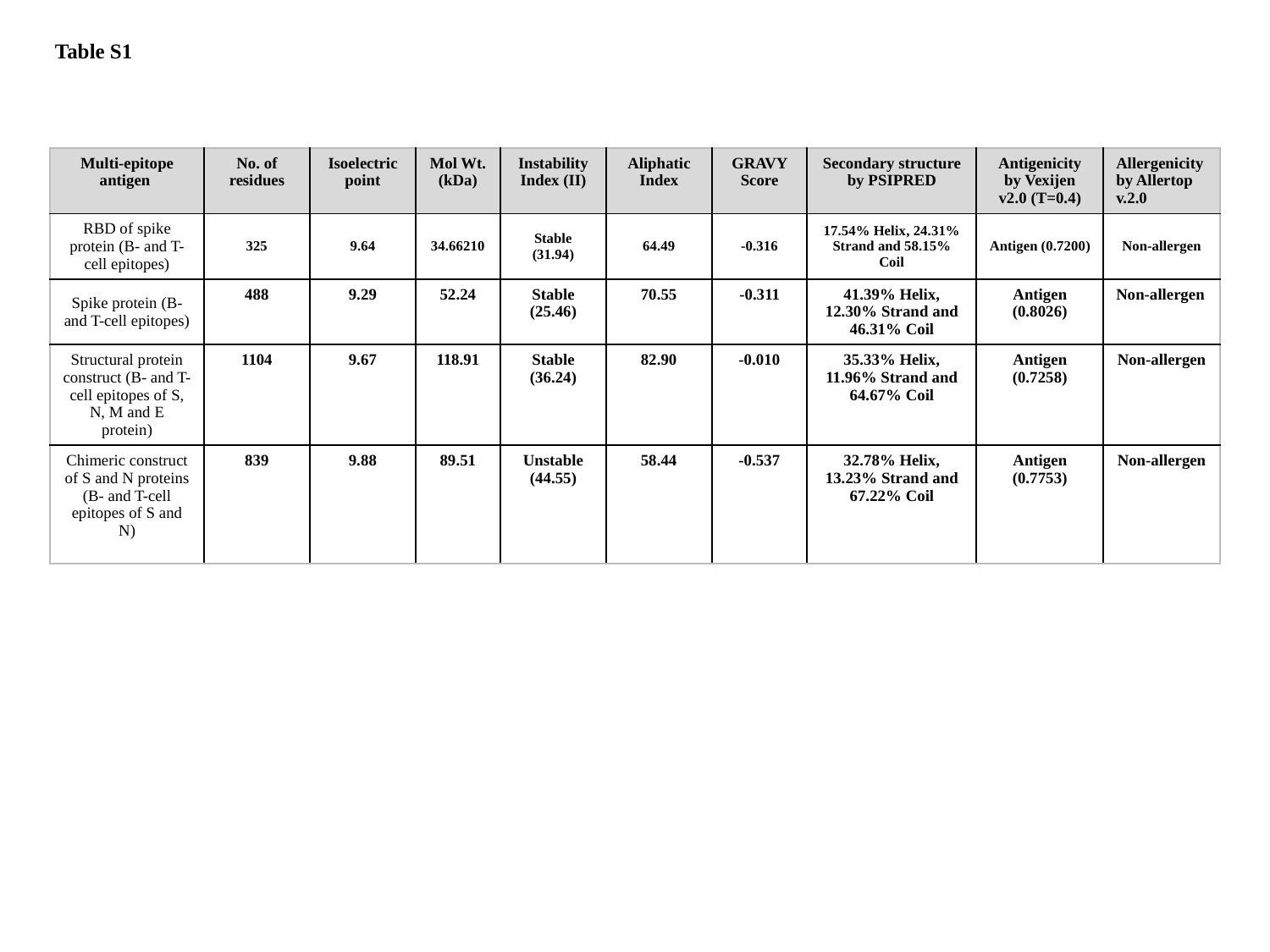

Table S1
| Multi-epitope antigen | No. of residues | Isoelectric point | Mol Wt. (kDa) | Instability Index (II) | Aliphatic Index | GRAVY Score | Secondary structure by PSIPRED | Antigenicity by Vexijen v2.0 (T=0.4) | Allergenicity by Allertop v.2.0 |
| --- | --- | --- | --- | --- | --- | --- | --- | --- | --- |
| RBD of spike protein (B- and T-cell epitopes) | 325 | 9.64 | 34.66210 | Stable (31.94) | 64.49 | -0.316 | 17.54% Helix, 24.31% Strand and 58.15% Coil | Antigen (0.7200) | Non-allergen |
| Spike protein (B- and T-cell epitopes) | 488 | 9.29 | 52.24 | Stable (25.46) | 70.55 | -0.311 | 41.39% Helix, 12.30% Strand and 46.31% Coil | Antigen (0.8026) | Non-allergen |
| Structural protein construct (B- and T-cell epitopes of S, N, M and E protein) | 1104 | 9.67 | 118.91 | Stable (36.24) | 82.90 | -0.010 | 35.33% Helix, 11.96% Strand and 64.67% Coil | Antigen (0.7258) | Non-allergen |
| Chimeric construct of S and N proteins (B- and T-cell epitopes of S and N) | 839 | 9.88 | 89.51 | Unstable (44.55) | 58.44 | -0.537 | 32.78% Helix, 13.23% Strand and 67.22% Coil | Antigen (0.7753) | Non-allergen |

## Slide 2
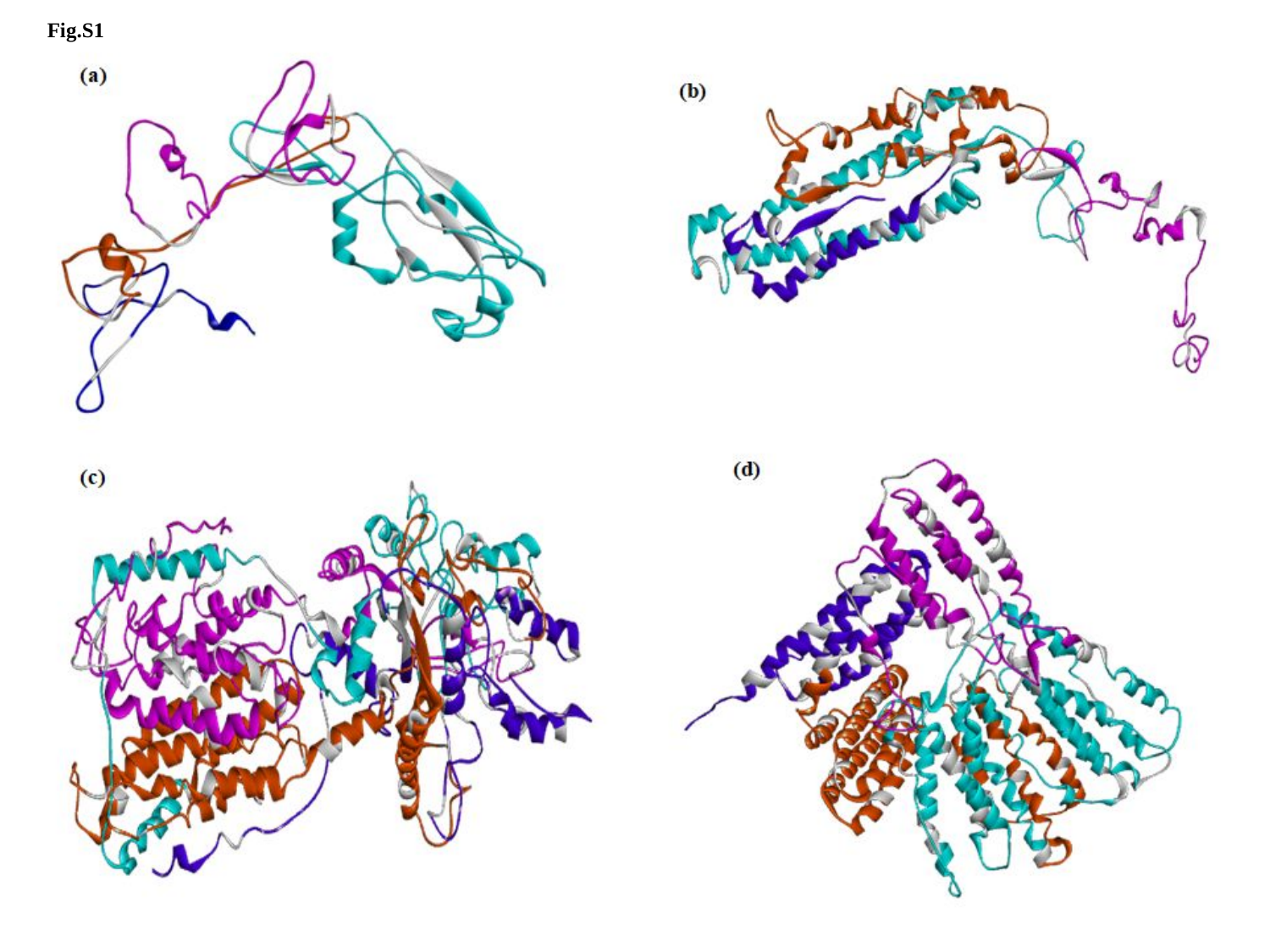

Fig.S1

## Slide 3
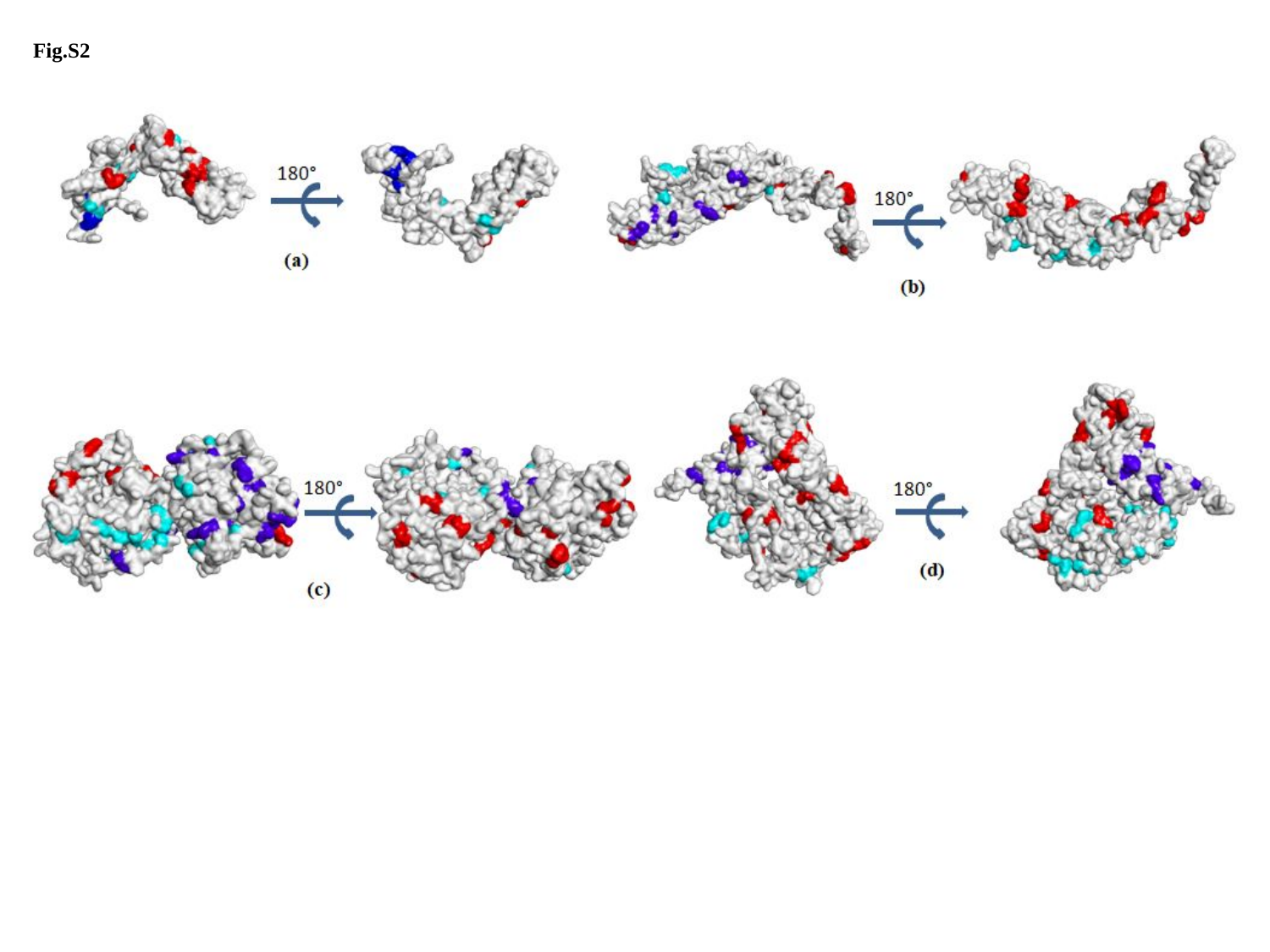

Fig.S2

## Slide 4
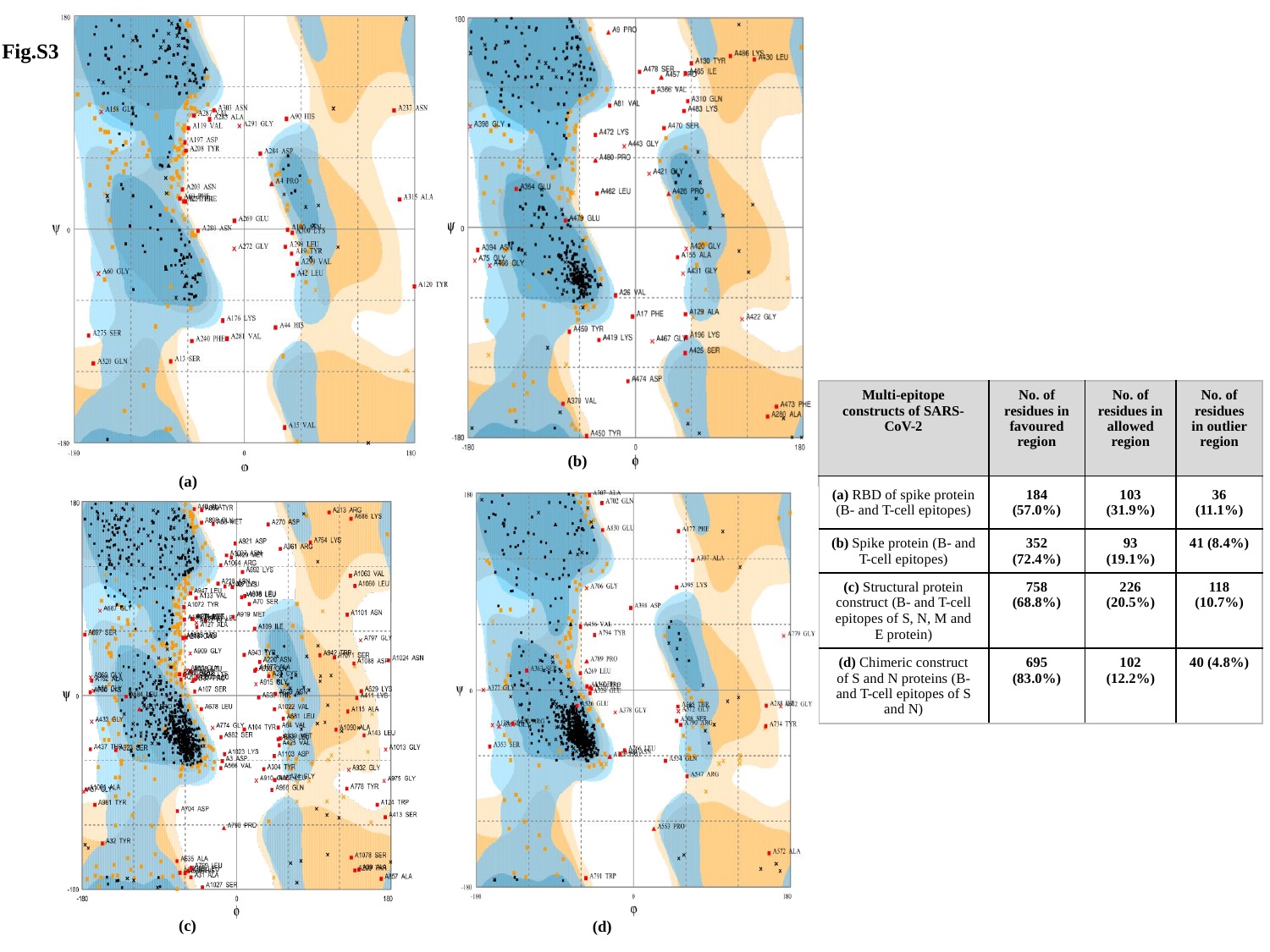

(b)
(a)
(c)
(d)
Fig.S3
| Multi-epitope constructs of SARS-CoV-2 | No. of residues in favoured region | No. of residues in allowed region | No. of residues in outlier region |
| --- | --- | --- | --- |
| (a) RBD of spike protein (B- and T-cell epitopes) | 184 (57.0%) | 103 (31.9%) | 36 (11.1%) |
| (b) Spike protein (B- and T-cell epitopes) | 352 (72.4%) | 93 (19.1%) | 41 (8.4%) |
| (c) Structural protein construct (B- and T-cell epitopes of S, N, M and E protein) | 758 (68.8%) | 226 (20.5%) | 118 (10.7%) |
| (d) Chimeric construct of S and N proteins (B- and T-cell epitopes of S and N) | 695 (83.0%) | 102 (12.2%) | 40 (4.8%) |

## Slide 5
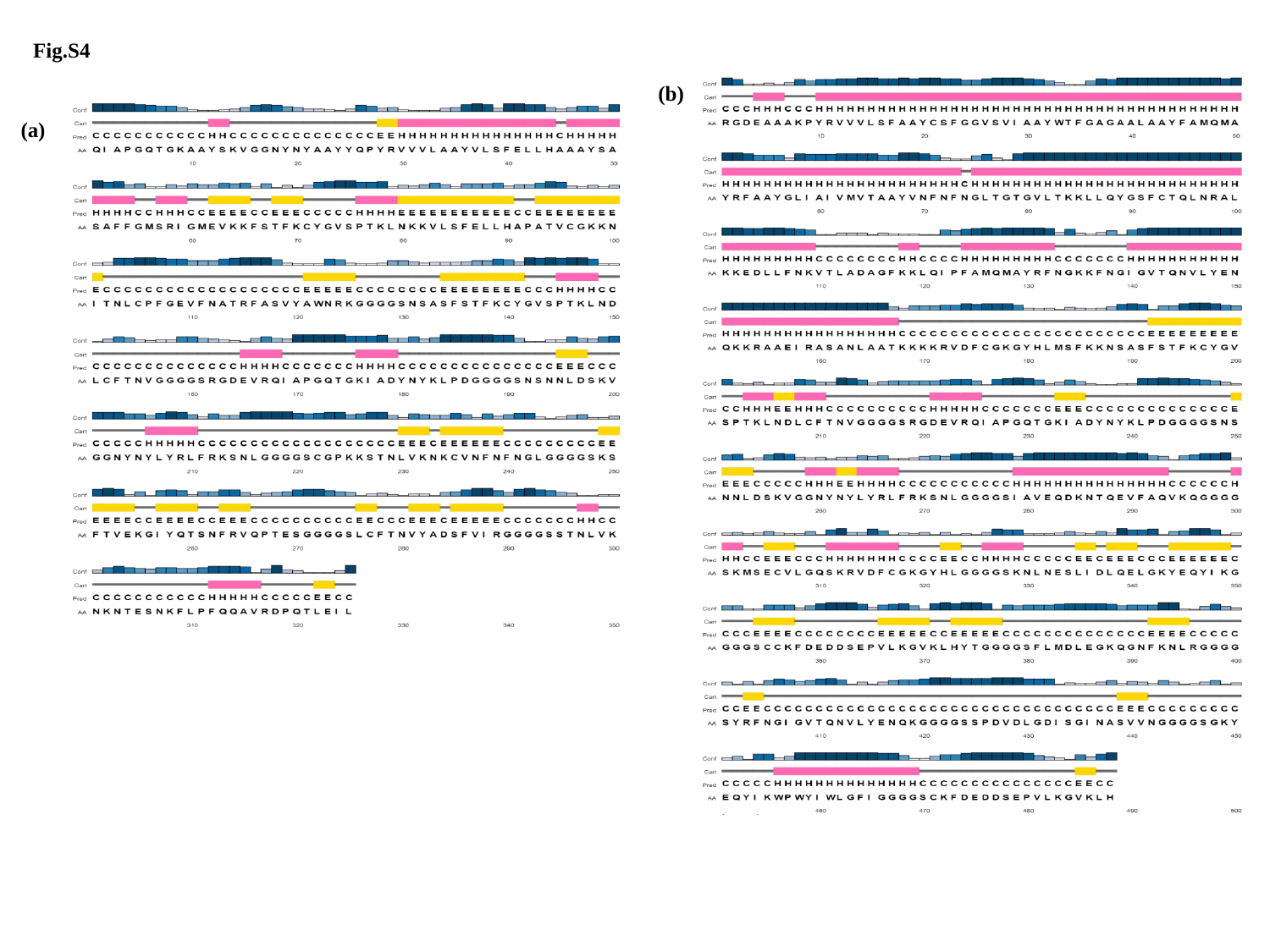

Fig.S4
(b)
(a)

## Slide 6
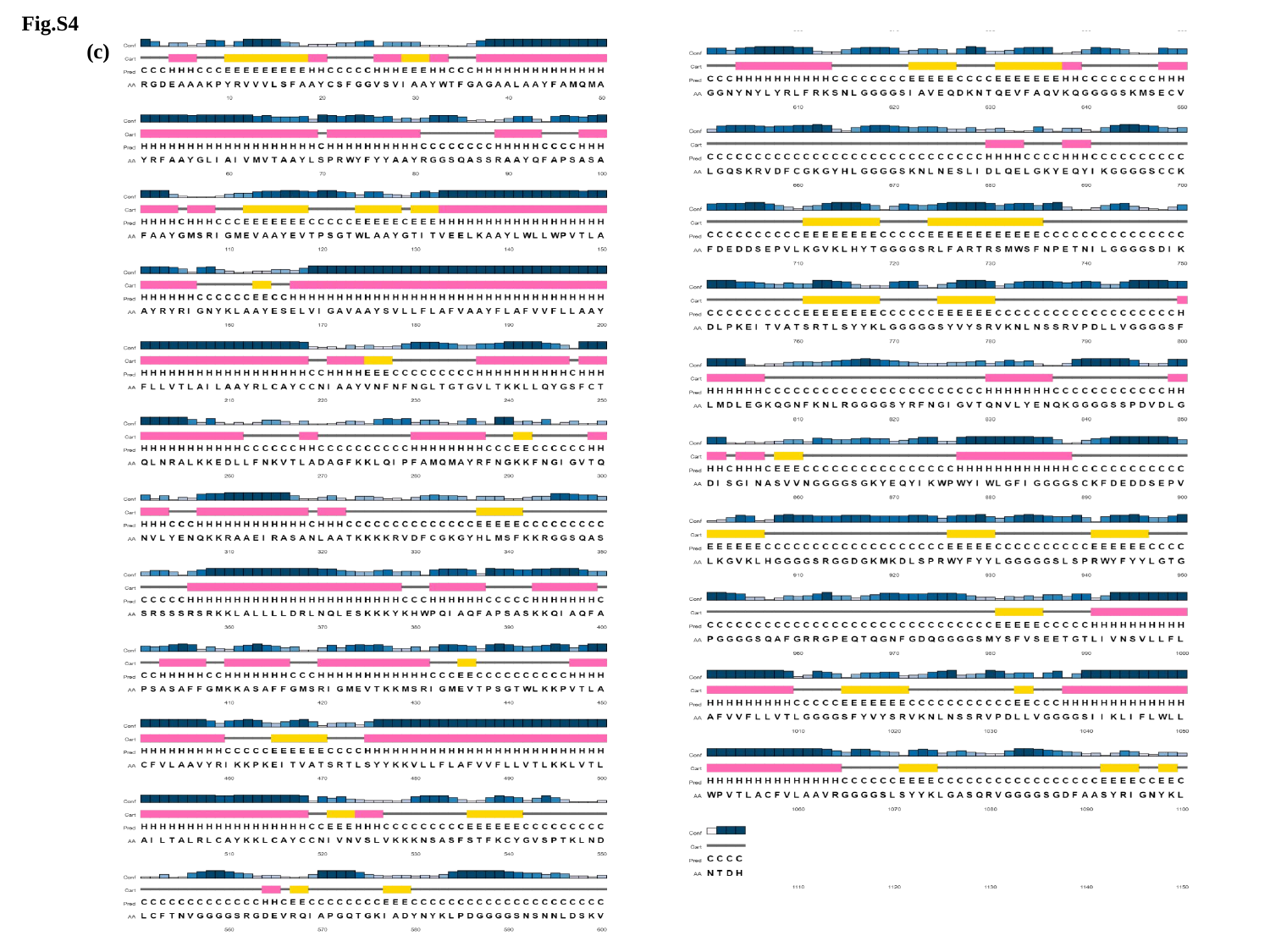

Fig.S4
(c)

## Slide 7
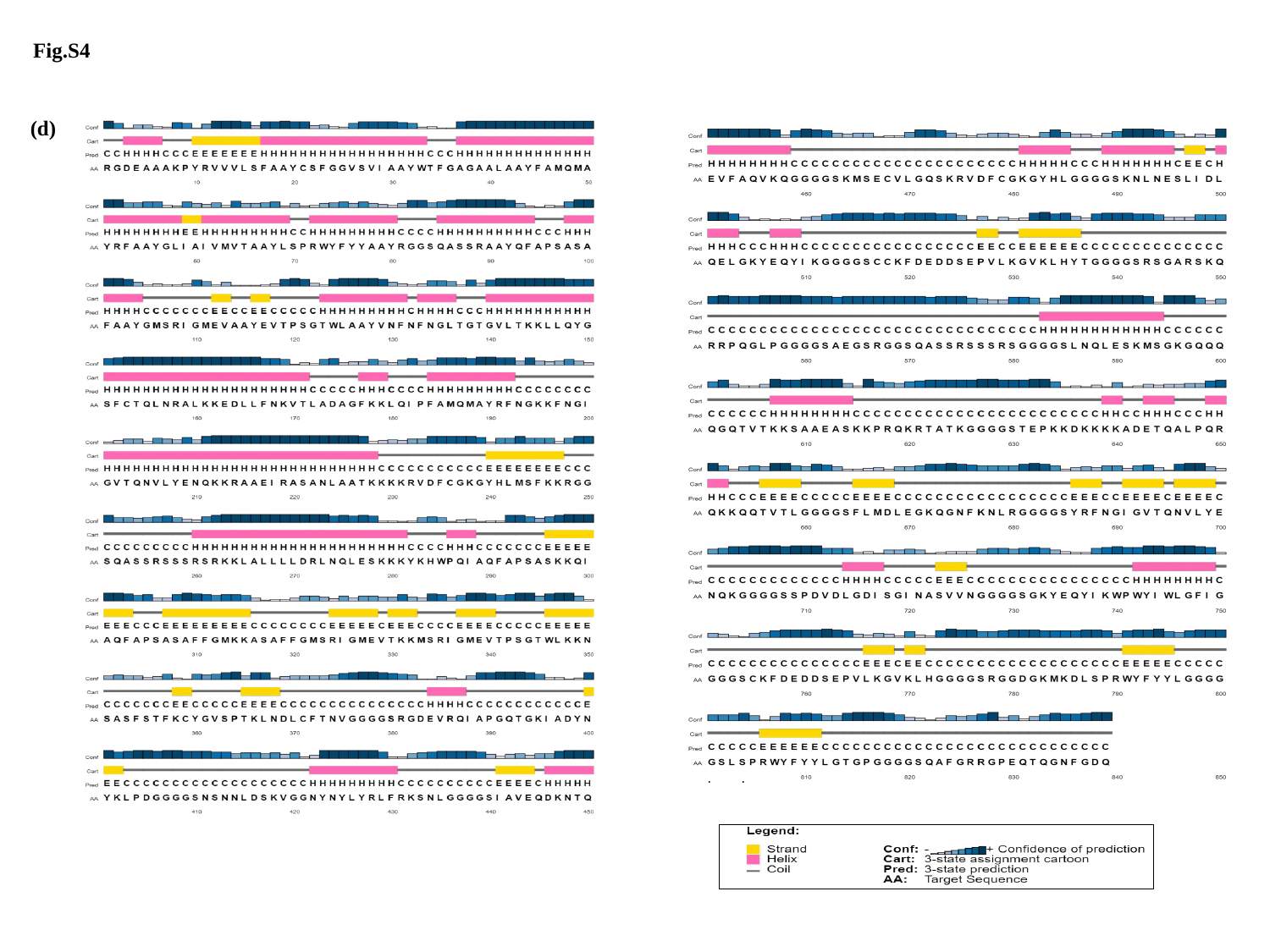

Fig.S4
(d)

## Slide 8
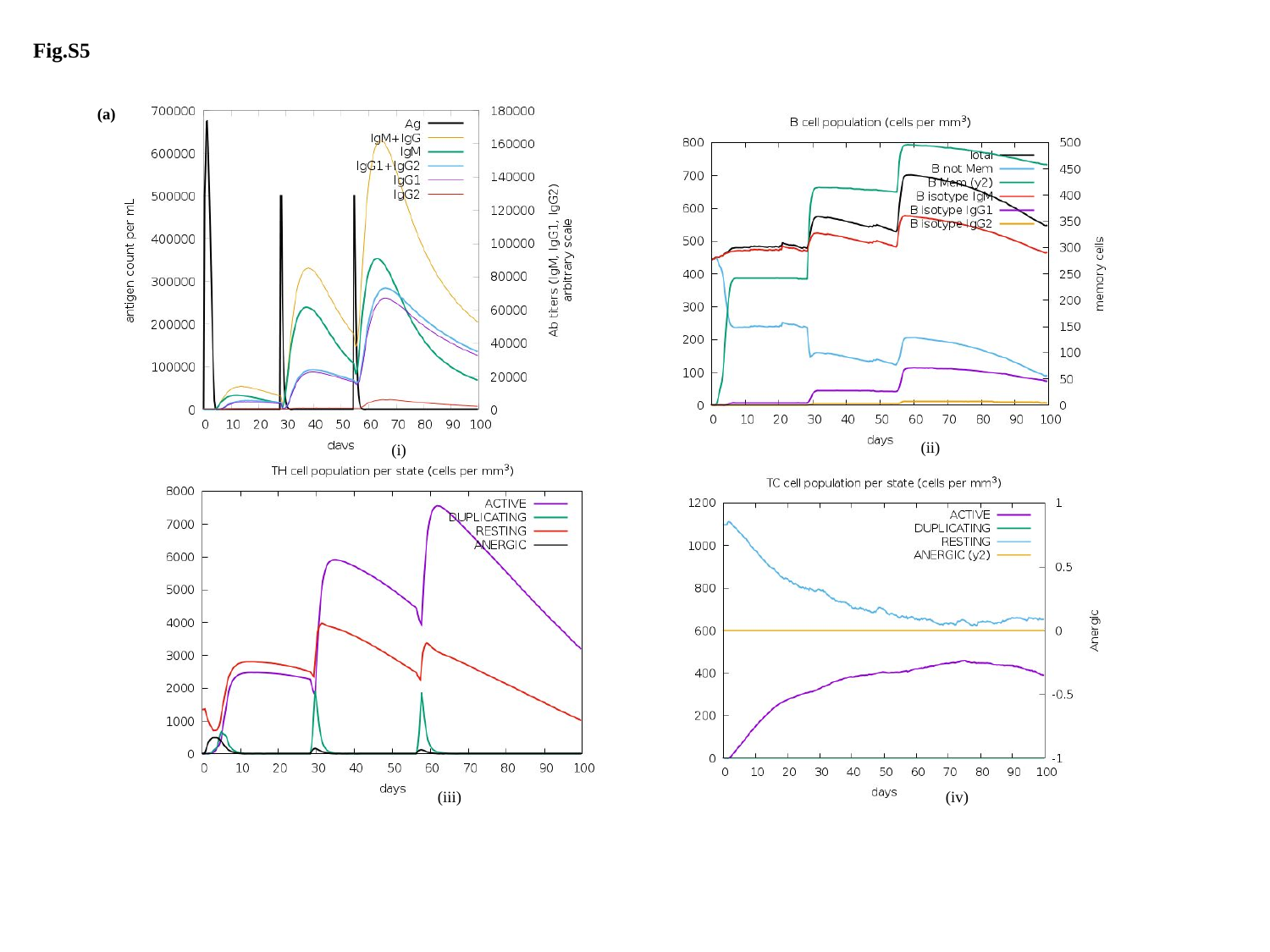

Fig.S5
(a)
(ii)
(i)
(iv)
(iii)

## Slide 9
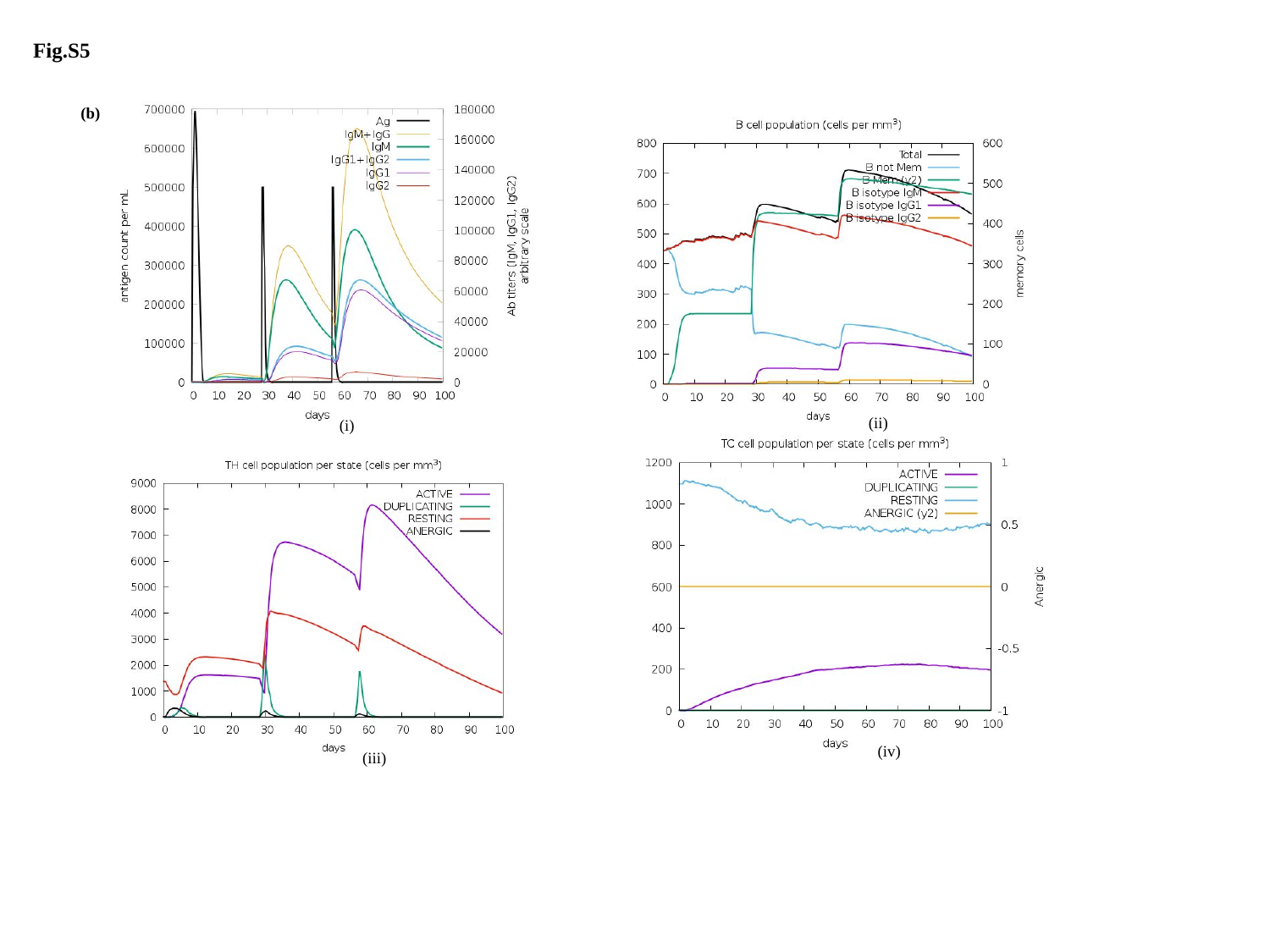

Fig.S5
(b)
(ii)
(i)
(iv)
(iii)

## Slide 10
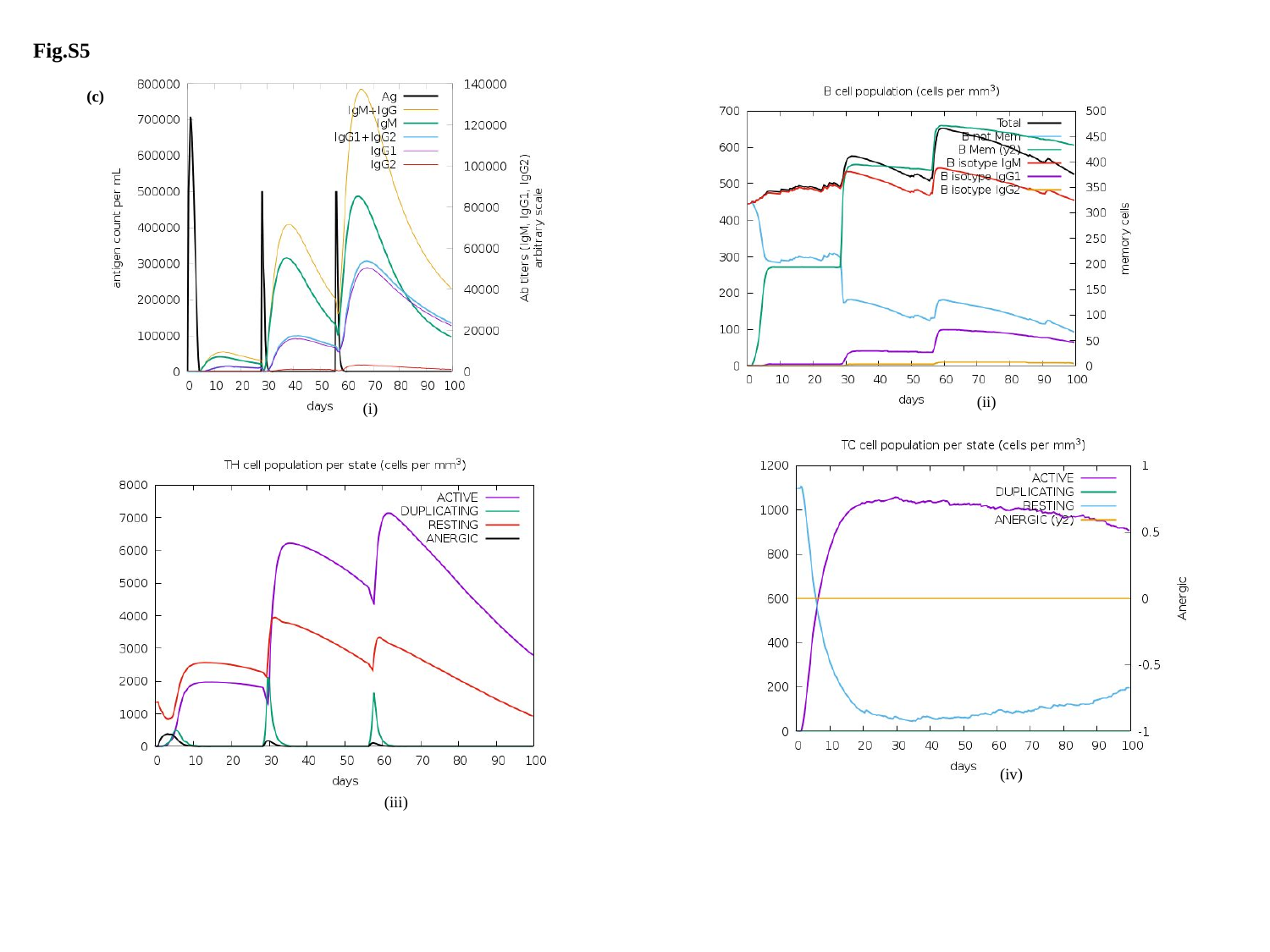

Fig.S5
(ii)
(i)
(iv)
(iii)
(c)

## Slide 11
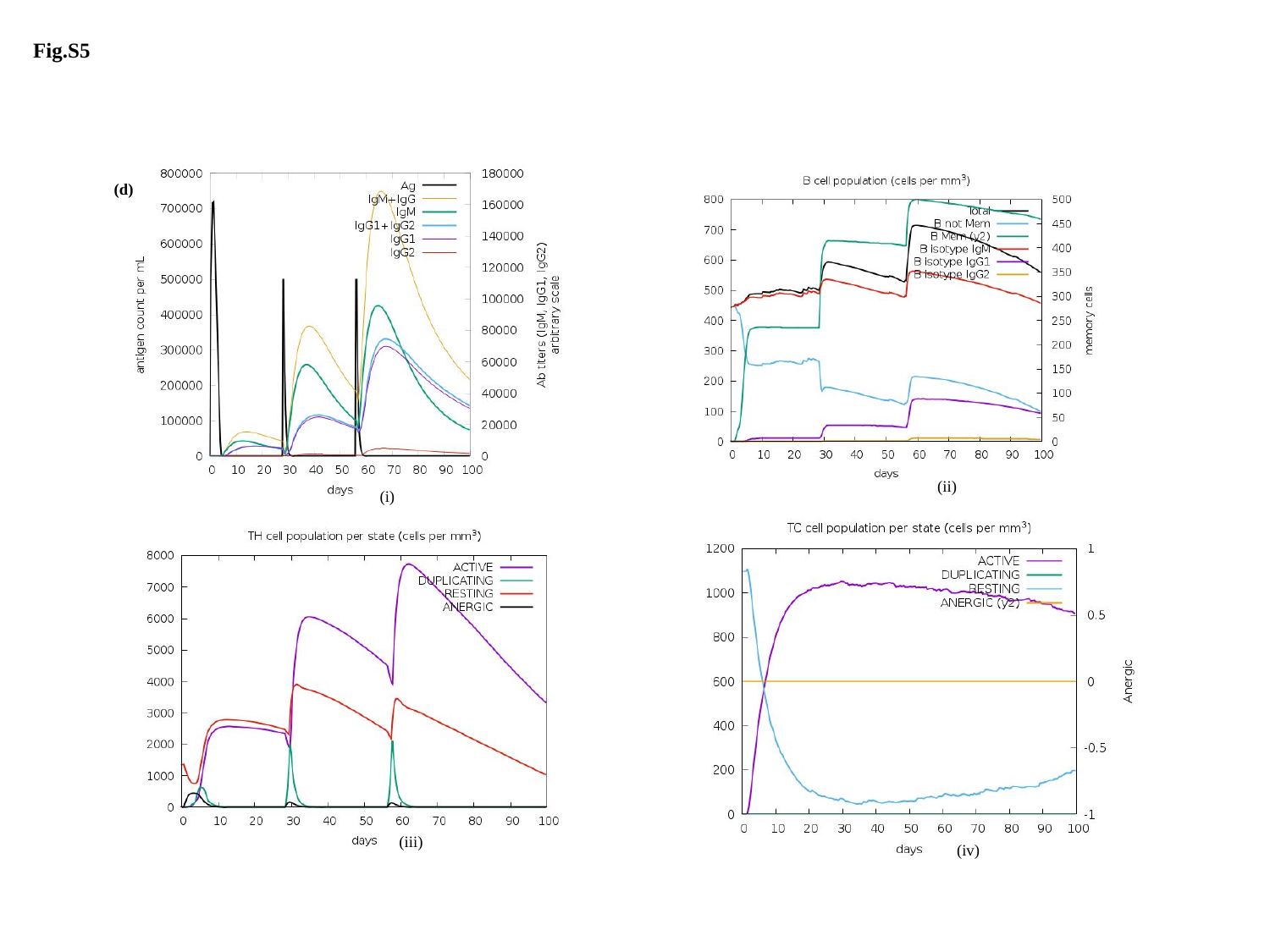

Fig.S5
(d)
(ii)
(i)
(iii)
(iv)
