# Supplementary material for: Immunoinformatics mapping of potential epitopes in SARS-CoV-2 structural proteins
Source: PLoS One. 2021 Nov 15;16(11):e0258645. doi: 10.1371/journal.pone.0258645 (PMC8592446; doi:10.1371/journal.pone.0258645)
Supplement: Attachment — Submitted filename: Response to Reviewers merged.pdf [file pone.0258645.s004.pdf]

**Journal Requirements:**

**Query 1.** Please review your reference list to ensure that it is complete and correct. If you have cited papers that have been retracted, please include the rationale for doing so in the manuscript text, or remove these references and replace them with relevant current references. Any changes to the reference list should be mentioned in the rebuttal letter that accompanies your revised manuscript. If you need to cite a retracted article, indicate the article's retracted status in the References list and also include a citation and full reference for the retraction notice.

**Response 1:** We sincerely acknowledged the suggestions. We now have revisited each of the cited and the listed references and corrected in the revised manuscript. Importantly, citation of reference 12 have been corrected, while reference 19, 71 and 72 have been replaced with relevant current references.

**Query 2: Additional Editor Comments (if provided):**
The English language writing quality is still not suitable for publication in PLOS ONE. Examples of the writing problems are provided by my suggested edits of the first page of the Introduction.
Line 67 deleted the comma
Line 68 add space after "NSP1."
delete comma after "ORF1b"
Line 70 replace "chymotrypsin- like" with "chymotrypsin-like"
Line 71 replace papain- like" with papain-like"
Line 76 delete comma
replace "and utilizes" with "while utilizing"
Line 85 replace ";." with "."
Line 86 replace "although a" with "A"
replace "T-cells response" with "T-cell responses"
Line 87 replace "epitopes, mainly" with "epitope"
Line 88 delete comma
delete "for"
Line 89 delete "vaccine"

**Response 2:** We are grateful to Academic Editor for pointing out the grammatical errors and mistakes. All the above-mentioned suggested corrections have now been incorporated in the revised manuscript.
**Query 3:** I found similar kinds of problems on reading the rest of the Introduction and the first two pages of the Discussion sections: mostly inappropriate use of commas and semicolons, some

misuse of singular and plural forms of nouns and verbs, incorrect presentation of a company name (BionTech vs BioNTech), failure to italicize et al., inappropriate word choice ("As on date" instead of "To date" line 481), proof reading failure (use of "previously at the beginning and end of a single sentence lines 474 and 476). These problems should be easily fixed by using commas and semicolons correctly and by careful proofreading.

**Response 3:** We greatly appreciate and duly acknowledged the suggestions of Academic Editor. Accordingly, we have taken the help of an English expert service providers to correct the grammar and the writing quality of our revised manuscript. All the suggested changes are highlighted in the revised manuscript with track changes. A certificate of editing is enclosed herewith for your kind information.

With warm regards,

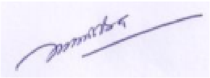

**(Nima D. Namsa)**
Corresponding author

# BLUE PENCIL ENGLISH LANGUAGE EDITING SERVICES

# CERTIFICATE OF EDITING

**This is to certify the manuscript specified below was edited by a highly qualified and experienced editor with native English fluency and usage.**

**Manuscript Title :**

**"Immunoinformatics mapping of potential epitopes in SARS-CoV-2 structural proteins"**

**Authors:**

*Yengkhom Damayanti Devi, Himanshu Ballav Goswami, Sushmita Konwar, Chandrima Doley, Anutee Dolley, Arpita Devi, Chen Chongtham, Dikshita Dowerah, Vashkar Biswa, Latonglila Jamir, Aditya Kumar, Siddhartha Shankar Satapathy, Suvendra Kumar Ray, Ramesh Chandra Deka, Robin Doley. Manabendra Mandal, Sandeep Das, Chongtham Shyamsunder Singh, Partha Pratim Borah, Pabitra Nath, and Nima D. Namsa**

**DATE ISSUED 23rd SEPTEMBER, 2021**

**CERTIFICATE VALIDATION KEY 117-20210239**

**Dr. Gaurangi Maitra, PhD, MNASc**

---

**Note: To validate the certificate please mail trobairitzg@gmail.com and quote certificate verification key.**
