# Supplementary material for: Immunoinformatics mapping of potential epitopes in SARS-CoV-2 structural proteins
Source: PLoS One. 2021 Nov 15;16(11):e0258645. doi: 10.1371/journal.pone.0258645 (PMC8592446; doi:10.1371/journal.pone.0258645)
Supplement: Attachment — Submitted filename: Response to Reviewers.doc [file pone.0258645.s002.doc]

A point-to-point rebuttal to comments of editor and reviewer is appended below for your kind information and perusal.

**Journal Requirements:**

When submitting your revision, we need you to address these additional requirements.

**Query 1.** Please ensure that your manuscript meets PLOS ONE's style requirements, including those for file naming. The PLOS ONE style templates can be found at

https://journals.plos.org/plosone/s/file?id=wjVg/PLOSOne_formatting_sample_main_body.pdf and https://journals.plos.org/plosone/s/file?id=ba62/PLOSOne_formatting_sample_title_authors_affiliations.pdf

**Response 1:** We thank profusely the academic editor. We have now utilized the templates and formatted the manuscript as per requirement of PLOS ONE.

**Query 2.** Please review your reference list to ensure that it is complete and correct. If you have cited papers that have been retracted, please include the rationale for doing so in the manuscript text, or remove these references and replace them with relevant current references. Any changes to the reference list should be mentioned in the rebuttal letter that accompanies your revised manuscript. If you need to cite a retracted article, indicate the article’s retracted status in the References list and also include a citation and full reference for the retraction notice.

**Response 2:** We now have revisited the list of references and appropriately cited all the reference in the text and the same is provided under reference list.

**Query 3.** Please add the tables into the manuscript.

**Response 3:** As suggested all the tables have now been included at the end of the main text of revised manuscript.

**Query 4.** We note that you have indicated that data from this study are available upon request. PLOS only allows data to be available upon request if there are legal or ethical restrictions on sharing data publicly. For more information on unacceptable data access restrictions, please see http://journals.plos.org/plosone/s/data-availability#loc-unacceptable-data-access-restrictions.

In your revised cover letter, please address the following prompts:

a) If there are ethical or legal restrictions on sharing a de-identified data set, please explain them in detail (e.g., data contain potentially sensitive information, data are owned by a third-party organization, etc.) and who has imposed them (e.g., an ethics committee). Please also provide contact information for a data access committee, ethics committee, or other institutional body to which data requests may be sent.

**Response 4a:** All the data associated with the manuscript is now provided as supplementary files.

b) If there are no restrictions, please upload the minimal anonymized data set necessary to replicate your study findings as either Supporting Information files or to a stable, public repository and provide us with the relevant URLs, DOIs, or accession numbers. For a list of acceptable repositories, please see http://journals.plos.org/plosone/s/data-availability#loc-recommended-repositories.

We will update your Data Availability statement on your behalf to reflect the information you provide.

**Response 4b:** All the data associated with the manuscript is now provided as supplementary files. We greatly appreciate the Journal team to update our Data Availability statement.

**Query 5.** We note that Figures 2 and 3 in your submission contain copyrighted images. All PLOS content is published under the Creative Commons Attribution License (CC BY 4.0), which means that the manuscript, images, and Supporting Information files will be freely available online, and any third party is permitted to access, download, copy, distribute, and use these materials in any way, even commercially, with proper attribution. For more information, see our copyright guidelines: http://journals.plos.org/plosone/s/licenses-and-copyright.

We require you to either (1) present written permission from the copyright holder to publish these figures specifically under the CC BY 4.0 license, or (2) remove the figures from your submission:

a. You may seek permission from the original copyright holder of Figures 2 and 3 to publish the content specifically under the CC BY 4.0 license.

We recommend that you contact the original copyright holder with the Content Permission Form (http://journals.plos.org/plosone/s/file?id=7c09/content-permission-form.pdf) and the following text:

“I request permission for the open-access journal PLOS ONE to publish XXX under the Creative Commons Attribution License (CCAL) CC BY 4.0 (http://creativecommons.org/licenses/by/4.0/). Please be aware that this license allows unrestricted use and distribution, even commercially, by third parties. Please reply and provide explicit written permission to publish XXX under a CC BY license and complete the attached form.”

Please upload the completed Content Permission Form or other proof of granted permissions as an "Other" file with your submission.

In the figure caption of the copyrighted figure, please include the following text: “Reprinted from [ref] under a CC BY license, with permission from [name of publisher], original copyright [original copyright year].”

b. If you are unable to obtain permission from the original copyright holder to publish these figures under the CC BY 4.0 license or if the copyright holder’s requirements are incompatible with the CC BY 4.0 license, please either i) remove the figure or ii) supply a replacement figure that complies with the CC BY 4.0 license. Please check copyright information on all replacement figures and update the figure caption with source information. If applicable, please specify in the figure caption text when a figure is similar but not identical to the original image and is therefore for illustrative purposes only.

**Response 5:** We greatly appreciate and respect the comments of Academic Editor with regard to copyrighted images of Figures 2 and 3 and we have taken this suggestion with utmost care. The figures represented structures of SARS-CoV-2 structural proteins (S, E, M & N) which were retrieved from RCSB PDB and homology modelling was carried out by our research group. The structures were represented in surface model in Biovia Discovery studio 2017 R2 software package and the predicted B-cell epitopes were represented by different colors. The epitopes mapped on the surface were predicted using the multiple computational resources by our team and the findings are outcome of our detailed investigations.

As suggested by the Academic Editor, we have redrawn the figure using structure of E protein with colors representing the predicted B cell epitopes reported in this manuscript. Biovia Discovery studio 2017 R2 software package have been used and the figure is provided below for your kind information and perusal. In this regard, I solicit you to reconsider this issue and provide your suggestions that might have contributed to the copyright issue. Appropriate references have been included.

**Fig.** E protein of SARS-CoV-2

**Reviewer’s Comments:**

**Comments to the Author**

1. Is the manuscript technically sound, and do the data support the conclusions?
The manuscript must describe a technically sound piece of scientific research with data that supports the conclusions. Experiments must have been conducted rigorously, with appropriate controls, replication, and sample sizes. The conclusions must be drawn appropriately based on the data presented.

Reviewer #1: Yes

2. Has the statistical analysis been performed appropriately and rigorously?

Reviewer #1: N/A

3. Have the authors made all data underlying the findings in their manuscript fully available?

The PLOS Data policy requires authors to make all data underlying the findings described in their manuscript fully available without restriction, with rare exception (please refer to the Data Availability Statement in the manuscript PDF file). The data should be provided as part of the manuscript or its supporting information, or deposited to a public repository. For example, in addition to summary statistics, the data points behind means, medians and variance measures should be available. If there are restrictions on publicly sharing data—e.g. participant privacy or use of data from a third party—those must be specified.

Reviewer #1: Yes

4. Is the manuscript presented in an intelligible fashion and written in standard English?

PLOS ONE does not copyedit accepted manuscripts, so the language in submitted articles must be clear, correct, and unambiguous. Any typographical or grammatical errors should be corrected at revision, so please note any specific errors here.

Reviewer #1: Yes

5. Review Comments to the Author

Please use the space provided to explain your answers to the questions above. You may also include additional comments for the author, including concerns about dual publication, research ethics, or publication ethics. (Please upload your review as an attachment if it exceeds 20,000 characters)

**Reviewer #1:** Devi and colleagues use a bioinformatic pipeline utilizing several platforms to predict B and T cell epitopes of several structural proteins (S, E, M and N) of SARS-CoV-2. The overall goal is to use this method to predict epitopes that can help guide the development of vaccines that are immunogenic, non-allergenic, and cytokine inducing. It can also present potential target for further validation for experimental work. The manuscript is generally well written, but should also address the pitfalls of the type of epitopes chosen. For example, while linear B-cell epitopes can arguably be easily identified using different approaches, it is unclear how these can elicit protective antibodies. It is difficult to design vaccines based off of linear epitopes (and arguably the same goes for conformational epitopes) – the challenge is whether expressing these epitopes on their own will truly recapitulate its native antigenic structure as a subunit vaccine. These hurdles should be discussed. In comparison, T cell epitopes are generally displayed as linear epitopes on the surface of MHC molecules and may not suffer the challenges of B cell epitopes.

**Response:** We profusely appreciate the suggestions of anonymous reviewer. Accordingly, we have added few lines on the above under the head_Discussion part of revised manuscript and the same is provided as follows:

In this study we designed a multi-epitope chimeric constructs that included the predicted B- and T-cell epitopes of the S, E, M and N proteins of SARS-CoV-2. Some of the epitopes predicted in this study have been verified experimentally by others. We included CTL, HTL and B-cell predicted epitopes in the chimeric constructs and designing efficacious multi-epitope vaccines remains a great challenge. Expression of the recombinant multi-epitope protein from the constructed multi-epitope vaccine gene and their ability to retain native antigenic structure as a subunit vaccine to elicit protective immune responses remains a major hurdle. In addition, it is unclear whether the linear and conformational B-cell epitopes designed to elicit a B cell will properly fold into conformations resembling the native S, E, M and N proteins to elicit a protective immune response. Tembusu virus (TMUV) is a newly emerging flavivirus that caused rapid egg drop and neurological symptoms in ducks. Potential *in-silico* predicted B-cell epitopes of envelope protein of TMUV fused with Glutathione S-transferases tag have been successfully expressed in *E. coli* [88]. The findings suggested that two of four predicted B cell epitopes could elicit neutralizing antibodies in ducks and provided protection against TMUV challenge [88]. A chimeric recombinant antigen comprising of predicted CD4+ and CD8+ T cell-specific epitopes derived from four *Leishmania infantum* proteins was successfully expressed and the purified antigen showed protective efficacy in mice against *Leishmania amazonensis* infection [89]. Thus, the present bioinformatic prediction provides signals for the design of potential synthetic peptides and its validation using convalescent sera of COVID-19 patients. However, it remains to be investigated whether a pool of B- and T-cell epitopes identified in this work can stimulate B- and T-cells by measuring the amount of released cytokines both *in vitro* and *in vivo*, thus providing a platform for future investigations.

1. The N protein is not expected to elicit neutralizing antibodies as it is a viral protein not expressed on the surface of viral particles. Only the S has been demonstrated to elicit neutralizing antibodies. While there may be reports of N neutralizing antibodies, it is generally accepted in the field that antibodies that target the nucleoprotein is not neutralizing. Please rephrase any statements describing neutralizing antibodies that target the N protein.

**Response:** We profusely appreciate the suggestions of anonymous reviewer. Accordingly, we have rephrased all statements describing neutralizing antibodies that target the N protein throughout the text of revised manuscript.

2. There are more than 3 different COVID vaccines that have received emergency use approval around the world depending on which country the authors are referring to – please clarify and correct.

**Response:** As suggested by the expert, we have re-written and provide all the details of COVID-19 vaccines (AstraZeneca/Oxford vaccine, Moderna, Johnson and Johnson, Pfizer/BionTech, Sinopharm, and Sinovac) which are recommended by WHO for emergency use. New references have been added from reference no. 19 to 26 under_ Introduction part of revised manuscript.

3. Similarly, there are other published studies on phase 1 and 2/3 studies that reported on the safety and efficacy of other COVID vaccines other than the one the authors mentioned (ChAdOx1/AZD1222). If the authors note published studies regarding AZD1222, they should also mention ones from Moderna, Pfizer, Johnson and Johnson, etc.

**Response:** As suggested by the expert, we have re-written and provide all the details of COVID-19 vaccines (AstraZeneca/Oxford vaccine, Moderna, Johnson and Johnson, Pfizer/BionTech, Sinopharm, and Sinovac) which are recommended by WHO for emergency use. New references have been added from reference no. 19 to 26 under Introduction part of revised manuscript.

4. It is not clear what the authors are trying to state in the following statement: S and N proteins are the major targets for the development of vaccines and diagnostic reagents against SARS-CoV-2 due to extended time required for performing neutralization assays and the need for BSL-3 containment facility.

**Response:** As suggested by the expert, we have re-written the above sentence as ‘S and N proteins are the major targets for the development of vaccines and diagnostic reagents against SARS-CoV-2 using these recombinant antigens. Development of rapid antibody test requires the production of recombinant antigens and its validation using the serum of convalescent COVID-19 patients in an enzyme linked immunosorbent assay (ELISA). ELISA does not require the culture of SARS-CoV-2 virus in BSL-3 containment facility [10-14,67-69]’. This has been included in the appropriate text of revised manuscript.

5. The authors briefly mentioned conserved epitopes between SARS-CoV-1 and -CoV-2, but is not greatly discussed in the manuscript. Perhaps the authors should also put some emphasis on conserved epitopes between the two divergent viruses.

**Response:** We greatly appreciate the suggestions of anonymous reviewer. We now have discussed the predicted B and T cell epitopes that were common to both SARS-CoV and SARS-CoV-2 under the discussion part.

6. PLOS authors have the option to publish the peer review history of their article (what does this mean?). If published, this will include your full peer review and any attached files.

If you choose “no”, your identity will remain anonymous but your review may still be made public.

Do you want your identity to be public for this peer review? For information about this choice, including consent withdrawal, please see our Privacy Policy.

Reviewer #1: No

While revising your submission, please upload your figure files to the Preflight Analysis and Conversion Engine (PACE) digital diagnostic tool, <https://pacev2.apexcovantage.com/>.

**Response:** We have used and uploaded figures in the PACE as suggested.

With warm regards,

**(Nima D. Namsa)**

Corresponding author
