# Supplementary material for: Immunoinformatics mapping of potential epitopes in SARS-CoV-2 structural proteins
Source: PLoS One. 2021 Nov 15;16(11):e0258645. doi: 10.1371/journal.pone.0258645 (PMC8592446; doi:10.1371/journal.pone.0258645)
Supplement: Attachment — Submitted filename: Response to Reviewers.doc [file pone.0258645.s003.doc]

A point-to-point rebuttal to comments of Academic Editor is appended below for your kind information and perusal.

**Journal Requirements:**

**Query 1.** Please review your reference list to ensure that it is complete and correct. If you have cited papers that have been retracted, please include the rationale for doing so in the manuscript text, or remove these references and replace them with relevant current references. Any changes to the reference list should be mentioned in the rebuttal letter that accompanies your revised manuscript. If you need to cite a retracted article, indicate the article’s retracted status in the References list and also include a citation and full reference for the retraction notice.

**Response 1:** We gratefully acknowledged the suggestions. We now have revisited each of the cited and the listed references and corrected in the revised manuscript.

**Query 2: Additional Editor Comments (if provided):** I performed a careful reading of the abstract and introduction of the revised submission. My reading revealed to me that the manuscript requires an English language editorial pass to correct grammar and and other problems to bring the writing quality of the manuscript to the appropriate journal standards. I cannot be more specific on this issue nor can I review the entire submission as I cannot see line or page numbers in the manuscript and can thus not cite the location of any problems I might see in reviewing the revised submission. Please prepare a revised manuscript after the editorial pass and submit the manuscript with line and page numbers included.

**Response 2:** We greatly appreciate and duly acknowledged the suggestions of Academic Editor. Accordingly, we have taken the help of an English expert service providers to correct the grammar and the writing quality of our manuscript. All the suggested changes are highlighted in the revised manuscript file with track changes.

With warm regards,

**(Nima D. Namsa)**

Corresponding author
